# High cholesterol absorption efficiency increases the risk of the nonfatal and fatal atherosclerotic events

**DOI:** 10.1016/j.jlr.2025.100974

**Published:** 2025-12-30

**Authors:** Piia Simonen, Mitja Lääperi, Lotta Ulander, Juha Sinisalo, Helena Gylling

**Affiliations:** Heart and Lung Center, Cardiology, Helsinki University Hospital and University of Helsinki, Helsinki, Finland

**Keywords:** acute coronary syndrome, atherosclerotic cardiovascular diseases, cholesterol absorption, cholesterol synthesis, coronary artery disease, LDL cholesterol, phytosterols, noncholesterol sterols

## Abstract

The aim of this study was to investigate whether cholesterol metabolism, especially high cholesterol absorption, affects atherosclerotic event risk in acute coronary syndrome (ACS) versus control patients without coronary artery disease (CAD). We set up a randomized, case-control substudy of a Corogene cohort (total cohort 5,295 consecutive patients admitted for coronary angiography because of stable or atypical chest pains and followed up for a median of 9.5 [interquartile range, 8.9–10.0] years). Of these, 200 ACS patients were matched by sex, age, BMI, LDL-C, and serum triglycerides to 200 patients without CAD. Blood samples were available in 363 cases (study population: ACS, n = 168; no CAD, n = 195) for analysis of serum biomarkers of relative cholesterol metabolism. High cholesterol absorption was associated with nonfatal and fatal acute CAD events in the ACS group but not in the no CAD group, whereas good compliance with statin treatment during the follow-up and low LDL-C concentration associated with good prognosis. Patients with high cholesterol absorption showed the worst survival probability during the follow-up, whereas individuals with low cholesterol absorption showed the best survival probability. In conclusion, high cholesterol absorption was associated with nonfatal and fatal acute CAD events in the ACS group in this exploratory clinical study. Individuals with high cholesterol absorption had more atherosclerotic events during the follow-up period, and their survival rate was worse than that of those with low cholesterol absorption. With therapy lowering both cholesterol absorption and LDL-C concentrations, cholesterol metabolism can be modified to become less atherogenic.

## Graphical abstract

High cholesterol absorption efficiency increases the risk of the nonfatal or fatal atherosclerotic events. ACS, acute coronary syndrome; CAD, coronary artery disease; High absorber, high cholesterol absorption efficiency; LDL, LDL-cholesterol; Low absorber, low cholesterol absorption efficiency; TG, triglyceride.

**Figure:**
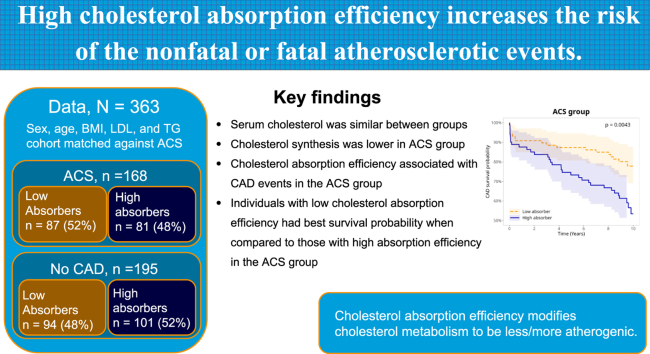

Atherosclerotic cardiovascular diseases (ASCVDs), especially coronary artery disease (CAD), are among the most common disease groups causing morbidity and mortality worldwide (1). The risk of CAD events has not diminished in time but is spreading in younger generations (1, 2). The development and progression of atherosclerosis can be inhibited by diminishing the amount of circulating LDL-lipoproteins and consequently LDL-C concentrations (3, 4, 5, 6).

Of the variables of whole-body cholesterol metabolism, high cholesterol absorption might have atherogenic potential (7, 8, 9, 10, 11). Cholesterol absorption is genetically determined (12, 13, 14, 15), and in line with the homeostasis of cholesterol metabolism, high cholesterol absorption is associated with low cholesterol synthesis, low reverse cholesterol transport from the tissues, and low biliary cholesterol elimination from the body to the feces (8, 9, 10, 11). In low cholesterol absorbers, cholesterol metabolism operates in the opposite direction, which may be protective with regard to the risk of atherosclerosis (8, 9, 10, 11, 14, 15). Cholesterol absorption efficiency ranges from 29% to 80% in general populations, and ≥50% is considered high (16, 17). Approximately 30% of individuals are estimated to be high cholesterol absorbers (16, 17).

The aim of this matched cohort substudy of a large “Genetic Predisposition of Coronary Artery Disease” cohort (Corogene) was to investigate the impact of cholesterol metabolism on the prevalence and event risks of ASCVDs, in particular among acute coronary syndrome (ACS) patients versus patients without CAD admitted to Helsinki University Central Hospital and followed up for a median of 9.5 (interquartile range [IQR], 8.9–10.0) years (18). We assumed that this study population, with and without ACS events at baseline and combined with a long follow-up period, would be optimal in demonstrating possible differences in atherogenicity of cholesterol metabolism and their potential consequences. Relative cholesterol metabolism was assessed by using validated serum biomarkers of cholesterol synthesis and cholesterol absorption efficiency (19, 20, 21). Our hypothesis was that differences in atherogenicity are related to cholesterol metabolism and especially to high cholesterol absorption.

## Materials and methods

### Study population

The original prospective Corogene study was aimed at collecting updated information on the present risk factors of CAD and the risk of CAD events during follow-up (18). The study population consisted of 5,295 consecutive adult patients of Finnish origin (3,379 men and 1,916 women), who were admitted to hospital for coronary angiography in 2006–2008 because of stable or atypical chest pains. They were followed up until 2016, at a median of 9.5 years. Of the study population, 2,294 patients had ACS, and of these, those with unstable angina pectoris (n = 227), non-ST-elevation myocardial infarction (n = 1137), and ST-elevation myocardial infarction (n = 726) comprised the study group. Patients who did not have CAD (n = 1,207) comprised the control group. The criteria for ACS were an episode of chest pain typical of ischemia, and >50% stenosis in at least one coronary artery. The criteria for no CAD were normal coronaries or nonsignificant obstruction in coronary arteries (≤50%).

For this substudy, 200 patients were randomly collected from the original study population with ACS and matched by sex, age, BMI, LDL-C, and serum triglycerides (TGs) to 200 patients without CAD, comprising the control group. Blood samples were available in 363 cases for analysis of serum biomarkers of relative cholesterol metabolism, and these cases comprised the study population (Graphical Abstract).

All patients provided written informed consent for the Corogene registry (18). Helsinki University Central Hospital Ethics Committee approved the research protocol for the Corogene study (registry numbers: 426/E5/05, 205/E0/2007, HUS/152/2016, and HUS/1203/2016). This study also complies with the 1964 Declaration of Helsinki and subsequent revisions.

### Study design

After hospital admission, coronary angiography was performed in all cases, and during this process, fasting blood samples were obtained from the arterial line for laboratory tests. Statin treatment (mainly simvastatin, atorvastatin, or rosuvastatin) was started after angiography in all cases. After discharge from the hospital, the patients were followed up using nationwide registers, including, for example, the Hospital Discharge Register, revealing potential new hospital admissions (22); Statistics Finland, revealing deaths and causes of death in the Finnish population (23); and the Finnish Prescription Register, revealing purchases of medication (24).

### Laboratory analyses

The essential laboratory measurements concerning normal health were analyzed by using routine standardized methods at the Diagnostic Center, Helsinki University Hospital Laboratories (Table 1). Serum and lipoprotein lipids were also analyzed at Helsinki University Hospital Laboratories using the accredited photometric and direct enzymatic methods.

**Table 1:** Baseline clinical characteristics in the study population and further divided into groups without CAD and with ACS

Variables	Study population, n = 363	No CAD, n = 195	ACS, n = 168	*P*	Missing (%)
Men/women, n (%)	187/176 (52/48)	97/98 (49/50)	90/78 (54/46)	0.528	
Age, years	62.1 ± 2.98	61.8 ± 1.23	62.4 ± 4.17	0.080	
Weight, kg	78.1 ± 17.5	79.2 ± 18.3	76.7 ± 16.5	0.173	2 (0.5)
BMI, kg/m^2^	26.9 ± 5.07	27.0 ± 5.28	26.7 ± 4.84	0.573	2 (0.5)
Atherosclerotic risk factors, n (%)					
Dyslipidemia	213 (59)	106 (54)	107 (64)	0.087	
Hypertension	214 (59)	114 (58)	100 (59)	0.915	
T1D or T2D	52 (14)	24 (12)	28 (17)	0.293	
Current smoker	117 (32)	78 (40)	39 (23)	<0.001	
Exsmoker	103 (29)	33 (17)	70 (42)	<0.001	
Essential standard laboratory measurements					
Blood hemoglobin, g/L	138 ± 15.7	139 ± 15.2	136 ± 16.0	0.036	37 (10)
Blood HbA1c, mmol/mol	6.33 ± 1.17	6.97 ± 1.62	6.22 ± 1.04	0.161	292 (80)
Plasma creatinine, μmol/L^a^	74 (64–87)	76 (66–93)	71 (62–83)	0.003	68 (19)
Serum TSH, mU/L^a^	1.93 (1.33–3.04)	1.93 (1.28–3.16)	1.93 (1.40–2.81)	0.980	165 (45)
Plasma ASAT, U/L^a^	38 (28–52)	33 (25–38)	42 (29–64)	0.020	276 (76)
Plasma NT-proBNP, ng/L^a^	366 (155–1377)	287 (131–905)	630 (226–1785)	<0.001	24 (7)
Plasma NT-proBNP, ≥600 ng/L	133 (39)	58 (30)	75 (51)	<0.001	
Plasma NT-proBNP, ≥900 ng/L	110 (32)	48 (25)	62 (42)	0.001	
Serum hs-CRP, mg/L^a^	2.3 (1.0–8.6)	1.6 (0.8–4.3)	4.2 (1.4–25.6)	<0.001	
Serum lipids, mmol/L					19 (5)
Serum cholesterol	4.84 ± 1.02	4.80 ± 1.04	4.89 ± 0.99	0.389	
LDL-C	2.92 ± 0.85	2.76 ± 0.81	3.13 ± 0.85	<0.001	
HDL-C	1.38 ± 0.45	1.47 ± 0.51	1.26 ± 0.34	<0.001	
Serum TGs	1.31 ± 0.62	1.27 ± 0.66	1.37 ± 0.57	0.140	
Statin treatment at baseline before angiography with good adherence, n (%)					
Statins, no	261 (72)	124 (64)	137 (82)		
Statins, yes	102 (28)	71 (36)	31 (18)	<0.001	
Statin treatment after angiography; good adherence for the whole follow-up period, n (%)					
Statins, no	221 (61)	149 (76)	72 (43)		
Statins, yes	142 (39)	46 (24)	96 (57)	<0.001	

Serum and lipoprotein lipids were analyzed enzymatically. Regarding the age of this study population, the optimal plasma cutoff of NT-proBNP diagnosing heart failure is >900 ng/L. Statin treatment: simvastatin, atorvastatin, or rosuvastatin.

Mean ± SD, n (%). Groups were compared using the *t-*test and chi^2^ test.

ASAT, aspartate aminotransferase; HbA1c, hemoglobin A1c; hs-CRP, high sensitive C-reactive protein; TSH, thyroid-stimulating hormone.

a Values shown are median (IQR) and tested using the Mann-Whitney *U* test.

Serum concentrations of cholesterol precursors, squalene, zymostenol, desmosterol, and lathosterol, cholestanol (a metabolite of cholesterol), and sitosterol, campesterol, and avenasterol (phytosterols), together also with total serum cholesterol, were quantified by using capillary GC-LC equipment (Agilent 7890GC System; Agilent Technologies, Wilmington, DE), with a 50-m long Ultra 2 capillary column (5% phenylmethyl siloxane), and flame ionization detection with 5α-cholestane as the internal standard (25). The cholesterol precursors, cholestanol, and the phytosterols are called noncholesterol sterols herein. The serum concentrations of the noncholesterol sterols and squalene were adjusted to that of serum cholesterol obtained from the same GC-LC run and expressed as ratios to cholesterol, for example, serum squalene:C. As ratios, the noncholesterol sterols and squalene are validated biomarkers of relative cholesterol metabolism (19, 20, 21). Cholesterol precursors depict relative cholesterol synthesis, and cholestanol and the phytosterols depict relative cholesterol absorption, which is called cholesterol absorption efficiency herein. A prerequisite for investigating cholesterol metabolism includes the requirement that the studies should be performed under stable living conditions and under a steady state of cholesterol metabolism.

### Statistical analyses

We present the data as counts and percentages per group, with means and SDs or medians and IQRs. Normality and homogeneity of variance assumptions were checked before further analyses, and some variables were logarithmically transformed. Continuous variables were tested by using Student’s *t-*test or Mann-Whitney *U* test, and categorical variables were tested by using the chi^2^ test. Spearman’s rank correlation coefficients were calculated. To further investigate the effects of cholesterol absorption efficiency and statin use on the ASCVD events, we created Kaplan-Meier plots for combined nonfatal and fatal CAD events and tested the differences between the groups by using the log-rank test. We also fitted Cox regression models with age at follow-up to investigate the effect of cholesterol absorption efficiency on combined nonfatal and fatal CAD outcomes in the no CAD and ACS groups. Sample-size calculation was based on *t-*test with defined significance levels (α = 0.05 and β = 0.20). Using these estimates, the preset statistical power of 80% was achieved. A two-sided *P* value of <0.05 was considered statistically significant. The analyses were performed by using IBM SPSS for Windows 22.0 software (IBM SPSS, Chicago, IL) and R software (R Foundation for Statistical Computing, Vienna, Austria), version 4.5.0, with packages ggplot2, survival, and survminer (26, 27) (https://CRAN.R-project.org/package=survivalR), (https://CRAN.R-project.org/package=survivalR).

## Results

### Study population

Of the 363 participants, 187 were men and 176 were women (Table 1). Their mean age was 62.1 (SD, 2.98) years (range, 42–69 years), and their mean BMI was 26.9 (SD, 5.1) kg/m^2^ (range, 15–55 kg/m^2^). Twenty-four percent of the participants were obese (BMI ≥ 30 kg/m^2^).

The study population was divided into individuals without CAD and with ACS based on baseline angiography (Table 1). Accordingly, 195 individuals did not have CAD, and they comprised the control group, called no CAD herein, whereas 168 patients belonged to the ACS group. Age, weight, BMI, and the distribution of gender did not differ between the no CAD group and the ACS group.

Of the atherosclerotic risk factors, dyslipidemia and hypertension were present in 59% of the study population, but diabetes (T1D or T2D) was present in only 14% (Table 1). The distribution of these risk factors was similar between genders and between the no CAD group and the ACS group. Current smokers were more frequent in the no CAD group versus the ACS group, but exsmokers showed the reverse (*P* < 0.001 for both).

Laboratory test results concerning the overall health of the study population were within reference values, except for the concentrations of plasma aspartate aminotransferase (ASAT), plasma N-terminal natriuretic peptide (NT-proBNP), and serum high-sensitive C-reactive protein (hs-CRP) (Table 1). The optimal value for plasma NT-proBNP is <300 ng/L in general populations, but since age affects its serum levels and regarding the age range of this study population (42–69 years), the optimal plasma cutoff diagnosing heart failure is >900 ng/l. Thus, 48 (25%) of the patients in the no CAD group and 62 (42%) in the ACS group had NT-proBNP values indicating heart failure (*P* < 0.001). Levels of blood hemoglobin and plasma creatinine were lower in the ACS group versus the no CAD group, whereas the levels of aspartate aminotransferase, NT-proBNP, and hs-CRP were higher in the ACS group versus the no CAD group.

Regarding the serum and lipoprotein lipids, the concentrations of serum cholesterol and TG did not differ between the no CAD group and ACS group (Table 1). LDL-C concentrations were higher in the ACS group than in the no CAD group, whereas those of HDL-C showed the reverse.

The distribution of the baseline clinical characteristics and the laboratory test results concerning the overall health between the low and high cholesterol absorber subgroups in the no CAD group versus the ACS group is presented in Supplemental Table S1. Briefly, in the low absorbers, the distribution of gender was slightly different between the no CAD group and ACS group, whereas in the high absorbers, age was slightly different between the no CAD group and ACS group. Most of the atherosclerotic risk factors and the laboratory tests were similar in the low absorbers between the no CAD group and ACS group and in the high absorbers between the no CAD group and ACS group. However, plasma NT-proBNP and serum hs-CRP levels were higher in the ACS group versus the no CAD group and both in the low and high absorbers.

Regarding the serum and lipoprotein lipids, the concentrations of serum cholesterol and TG did not differ between any of the groups, but LDL-C concentration was higher in the ACS group versus the no CAD group and in both the low and high absorbers, whereas HDL-C showed the opposite (Supplemental Table S1).

**Fig. 1:**
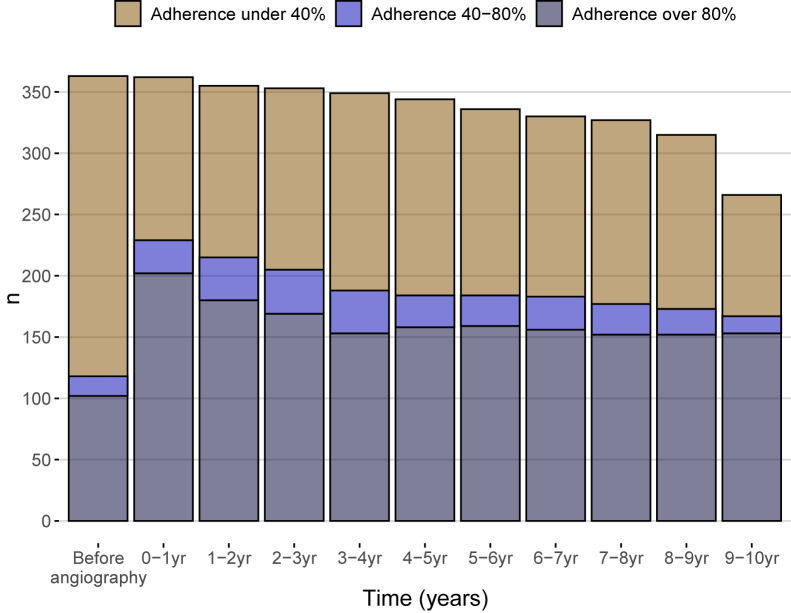
Statin adherence in the study population at baseline and during the 9.5-year follow-up. Statin adherence was considered to be high if the dose had been one tablet/day for 80% of the follow-up time. The median level of adherence from baseline to the end of the follow-up period was 58% (IQR, 5%–95%) in the whole study population.

Before hospital admission, 28% of the study population showed a good statin adherence rate of ≥80%, and 35% had an adherence rate of >0%, suggesting that they had used statin at least to some extent, but the frequency of adherence ≥80% was lower in the ACS group versus the no CAD group (18% vs. 36%, *P* < 0.001) (Table 1, Fig. 1). After angiography, 39% of the individuals had a statin adherence rate of ≥80% (24% in the no CAD group and 57% in the ACS group; *P* < 0.001).

### Cholesterol metabolism

Inverse correlations between the serum biomarkers of cholesterol synthesis and absorption, for example, the lathosterol:C and cholestanol:C ratios (r = −0.39; 95% CI = −0.48, −0.29; *P* < 0.001) indicated that the study population was metabolically in a steady state, and the results obtained by assay of the serum biomarkers of cholesterol metabolism could be considered valid. The serum biomarkers of cholesterol synthesis correlated with each other, as did the serum biomarkers of cholesterol absorption efficiency (*P* < 0.001 for both groups), confirming their appropriateness.

The biomarkers of cholesterol synthesis (except for desmosterol) were significantly higher in the no CAD group versus the ACS group (from *P* = 0.005 to *P* < 0.001) (Table 2). The biomarkers of cholesterol absorption efficiency were similar in the two groups.

**Table 2:** Concentrations of serum and lipoprotein lipids and serum noncholesterol sterol ratios to cholesterol in the study population without CAD and with ACS and further divided into subgroups of low and high cholesterol absorbers

Variables	No CAD, n = 195				ACS, n = 168				No CAD vs. ACS		
	All, n = 195	Low absorbers , n = 94	High absorbers, n = 101	*P*, n = 94 vs. 101	All, n = 168	Low absorbers, n = 87	High absorbers, n = 81	n = 87 vs. 81	All	Low absorbers	High absorbers
									*P*		
Serum cholesterol^1^	4.80 ± 1.04	4.89 ± 0.95	4.71 ± 1.11	0.205	4.89 ± 0.99	4.95 ± 0.89	4.83 ± 1.09	0.462	0.388	0.700	0.469
LDL-C^a^	2.76 ± 0.81	2.79 ± 0.71	2.74 ± 0.90	0.685	3.13 ± 0.85	3.21 ± 0.80	3.03 ± 0.90	0.187	<0.001	<0.001	0.039
HDL-C^a^	1.47 ± 0.51	1.52 ± 0.54	1.43 ± 0.48	0.227	1.26 ± 0.34	1.25 ± 0.29	1.27 ± 0.38	0.716	<0.001	<0.001	0.018
Serum TGs^a^	1.27 ± 0.66	1.36 ± 0.71	1.18 ± 0.60	0.061	1.37 ± 0.57	1.37 ± 0.49	1.37 ± 0.65	0.998	0.140	0.954	0.061
Serum biomarkers of cholesterol synthesis^b^											
Squalene:C	9.8 (7.5–14)	10 (7.5–14)	9.8 (7.7–14)	0.359	8.6 (6.3–12)	8.5 (6.8–12)	8.9 (5.8–12)	0.826	0.005	0.024	0.082
Zymostenol:C	36 (22–64)	54 (28–76)	27 (20–45)	<0.001	20 (13–36)	21 (15–40)	18 (12–34)	0.083	<0.001	<0.001	<0.001
Desmosterol:C	86 (74–108)	95 (79–110)	81 (69–103)	0.042	83 (72–100)	83 (72–101)	83 (71–98)	0.832	0.135	0.018	0.797
Lathosterol:C	86 (60–140)	117 (69–173)	73 (53–111)	<0.001	74 (50–112)	91 (61–133)	59 (45–92)	<0.001	0.003	0.010	0.040
Serum biomarkers of cholesterol absorption efficiency^b^											
Cholestanol:C	158 (137–187)	136 (126–145)	186 (169–228)	<0.001	155 (141–185)	141 (130–148)	185 (171–219)	<0.001	0.855	0.237	0.990
Sitosterol:C	120 (86–162)	97 (73–136)	146 (105–208)	<0.001	115 (85–156)	98 (78–122)	144 (111–194)	<0.001	0.648	0.562	0.836
Campesterol:C	219 (168–305)	190 (147–238)	269 (201–336)	<0.001	234 (165–297)	197 (148–249)	272 (219–351)	<0.001	0.710	0.603	0.637
Avenasterol:C	35 (28–45)	33 (25–39)	41 (31–51)	<0.001	35 (27–43)	31 (25–37)	39 (31–49)	<0.001	0.152	0.308	0.414

Values are mean ± SD or median (IQR). Groups were compared using the *t-*test and Mann-Whitney *U* test. Serum and lipoprotein lipids were analyzed enzymatically, but serum cholesterol, together with noncholesterol sterols, was analyzed by gas-liquid chromatography. Levels of noncholesterol sterols were assessed on a log_10_ scale. The study population was divided into two groups, without CAD and with ACS, and further into low and high cholesterol absorber subgroups based on the median value of the serum cholestanol:C ratio at baseline. Serum cholestanol:C ratio <157.3 10^2^ μmol/mmol of cholesterol depicts low and ≥157.3 10^2^ μmol/mmol of cholesterol high cholesterol absorption efficiency.

a mmol/L.

b 10^2^ μmol/mmol of cholesterol.

**Fig. 2:**
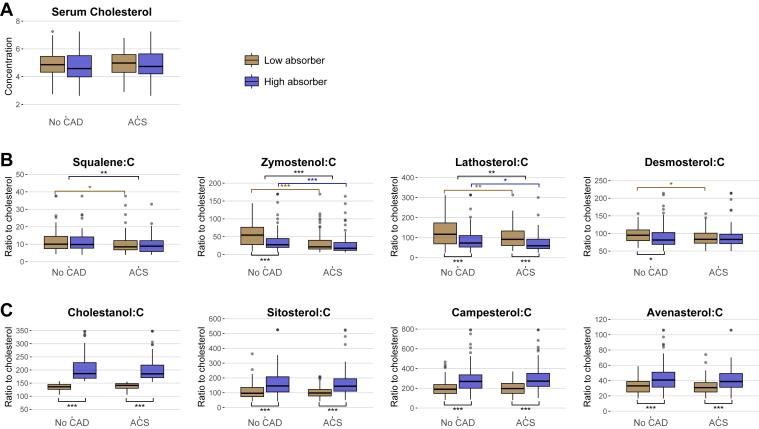
Box plots showing concentrations of serum cholesterol and the noncholesterol sterol ratios to cholesterol in the study population without CAD and with ACS and further divided into low and high cholesterol absorber subgroups. Groups were compared using a *t-*test (serum cholesterol synthesis and cholesterol absorption efficiency markers were logarithmized with a log10 scale). A: serum cholesterol; B: serum biomarkers of cholesterol synthesis; C: serum biomarkers of cholesterol absorption efficiency. The box shows the IQR, and the black line within the box shows the median. The whiskers show observations 1.58 ∗ IQR/sqrt(n), and observations beyond that are shown with points (i.e., outliers). Values were winsorized to 1st and 99th percentiles to avoid outliers in the plots. ∗*P* < 0.05, ∗∗*P* < 0.01, and ∗∗∗*P* < 0.001.

The no CAD group and ACS group were further divided into low- and high-cholesterol absorber subgroups based on the median value of the serum cholestanol:C ratio at baseline (Table 2, Fig. 2). A serum cholestanol:C ratio of <157.3 10^2^ μmol/mmol of cholesterol depicted low cholesterol absorption efficiency (n = 181) and a ratio of ≥157.3 depicted high cholesterol absorption efficiency (n = 182). Serum cholesterol concentrations did not differ between these subgroups, but LDL-C concentration was significantly higher in the ACS group versus the no CAD group and also in the respective cholesterol absorber subgroups. HDL-C concentrations were opposite to those of LDL-C, being significantly higher in the no CAD group versus the ACS group. The biomarkers of cholesterol synthesis were higher in the no CAD group versus the ACS group and also in the low versus high absorbers in both groups (Table 2, Fig. 2). The biomarkers of cholesterol absorption efficiency were higher in the high versus low absorbers in both the no CAD group and the ACS group.

### Statin treatment and cholesterol metabolism

Since statin treatment interferes with cholesterol metabolism, the rate of adherence to the treatment in the study population was first assessed from baseline to the end of the study (Fig. 1). A good adherence rate was considered to be ≥80%, and the median rate of adherence from baseline to the end of follow-up was 58% (IQR, 5%–95%). During the follow-up, only 24% (n = 46) had good adherence for statin use in the no CAD group compared with 57% (n = 96) in the ACS group (*P* < 0.001) (Table 1).

**Fig. 3:**
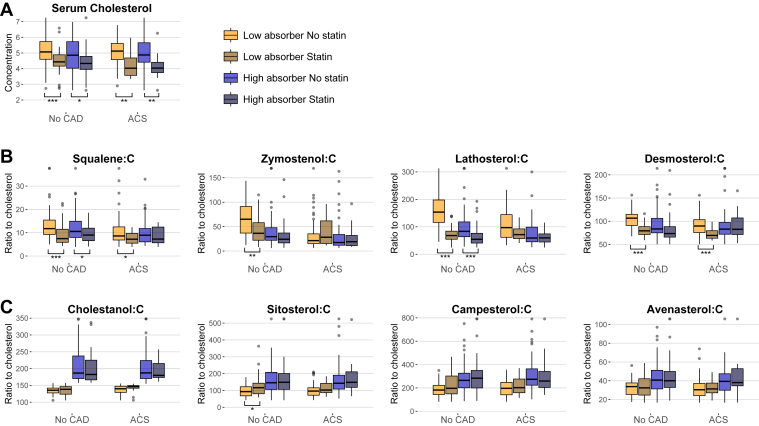
Box plots showing concentrations of serum cholesterol and the noncholesterol sterol ratios to cholesterol in the study population without CAD and with ACS and further divided into low and high cholesterol absorber subgroups without and with statin treatment. Groups were compared using *t-*test (serum cholesterol synthesis and cholesterol absorption markers were logarithmized with a log10 scale). A: serum cholesterol; B: serum biomarkers of cholesterol synthesis; C: serum biomarkers of cholesterol absorption efficiency. The box shows the IQR, and the black line within the box shows the median. The whiskers show observations 1.58∗ IQR/sqrt(n), and observations beyond that are shown with points (i.e., outliers). Values were winsorized to 1st and 99th percentilesto avoid outliers in the plots. ∗*P* < 0.05, ∗∗*P* < 0.01, and ∗∗∗*P* < 0.001.

Second, the effect of statin treatment on cholesterol metabolism was examined separately in the no CAD group and ACS group and in their respective cholesterol absorber subgroups (Table 3, Fig. 3). Statin treatment decreased serum cholesterol and LDL-C concentrations in the no CAD group and ACS group and in their cholesterol absorber subgroups. It also decreased the levels of the serum biomarkers of cholesterol synthesis in the no CAD low (from *P* = 0.003 to *P* < 0.001) and high absorbers (from *P* = 0.023 to *P* < 0.001), and in the ACS, low absorbers (from *P* = 0.046 to *P* < 0.001). However, statins had no effect on the biomarkers of cholesterol synthesis in the ACS high absorbers. Regarding the biomarkers of cholesterol absorption, the only effect of statins was that the sitosterol:C ratio increased in the no CAD-low-absorber subgroup (*P* = 0.011).

**Table 3:** Concentrations of serum and lipoprotein lipids and noncholesterol sterol ratios to cholesterol in the study population without CAD and with ACS and further divided into subgroups of low and high cholesterol absorbers and without and with statin treatment

Variables	No CAD, n = 195						ACS, n = 168					
	Low absorbers, n = 94			High absorbers, n = 101			Low absorbers, n = 87			High absorbers, n = 81		
	No statin, n = 59	Statin, n = 35	*P*	No statin, n = 65	Statin, n = 36	*P*	No statin, n = 74	Statin, n = 13	*P*	No statin, n = 63	Statin, n = 18	*P*
Serum cholesterol^a^	5.13 ± 0.94	4.49 ± 0.83	<0.001	4.87 ± 1.18	4.41 ± 0.93	0.031	5.09 ± 0.85	4.24 ± 0.81	0.003	5.00 ± 1.06	4.13 ± 0.88	0.004
LDL-C^a^	2.96 ± 0.68	2.49 ± 0.66	0.003	2.91 ± 0.91	2.44 ± 0.81	0.014	3.35 ± 0.74	2.55 ± 0.75	0.004	3.17 ± 0.89^c^	2.46 ± 0.71	0.009
HDL-C^a^	1.56 ± 0.62	1.44 ± 0.35	0.402	1.42 ± 0.48	1.44 ± 0.48	0.721	1.25 ± 0.30	1.25 ± 0.29	0.992	1.28 ± 0.38^c^	1.23 ± 0.38	0.651
Serum TGs^a^	1.42 ± 0.77	1.26 ± 0.60	0.300	1.14 ± 0.60	1.27 ± 0.60	0.244	1.39 ± 0.51	1.26 ± 0.33	0.458	1.38 ± 0.68	1.31 ± 0.54	0.832
Serum biomarkers of cholesterol synthesis^b^												
Squalene:C	11.8 (9.2–15.4)	7.5 (5.9–11.5)	<0.001	10.6 (8.0–14.9)	9.0 (6.6–11.9)	0.023	8.6 (6.9–12.5)^c^	7.2 (5.5–9.7)	0.046	9.0 (6.1–11.8)	7.3 (5.7–12.4)	0.226
Zymostenol:C	65.1 (36.8–91.1)	36.3 (22.1–58.0)	0.003	29.4 (22.1–47.9)	24.2 (16.6–36.9)	0.130	21.4 (15.2–35.2)^c^	28.2 (15.5–61.7)	0.286	17.5 (11.9–33.8)^c^	19.1 (11.1–32.0)	0.860
Lathosterol:C	153 (116–198)	68 (55–84)	<0.001	83 (64–118)	55 (41–76)	<0.001	97 (62–144)^c^	72 (57–92)	0.071	59 (45–99)^c^	60 (45–74)	0.243
Desmosterol:C	107 (91–115)	79 (72–88)	<0.001	83 (75–106)	73 (66–88)	0.055	90 (76–103)^c^	69 (63–80)	<0.001	83 (72–97)	83 (71–107)	0.960
Serum biomarkers of cholesterol absorption efficiency^b^												
Cholestanol:C	136 (128–142)	138 (124–146)	0.726	187 (170–238)	183 (166–225)	0.541	140 (129–147)	146 (142–150)	0.413	187 (170–224)	180 (173–215)	0.187
Sitosterol:C	93 (68–122)	116 (80–143)	0.011	146 (105–208)	148 (106–200)	0.747	96 (72–115)	102 (87–142)	0.116	142 (108–190)	148 (118–211)	0.591
Campesterol:C	182 (143–222)	197 (149–302)	0.103	265 (201–326)	283 (200–350)	0.743	197 (141–247)	202 (159–276)	0.341	274 (224–363)	259 (204–341)	0.963
Avenasterol:C	33.8 (25.5–37.7)	32.5 (24.7–42.1)	0.996	40.8 (31.3–51.2)	40.0 (32.9–49.9)	0.691	30.4 (24.1–37.3)	31.3 (27.2–37.6)	0.451	39.3 (29.9–47.5)	38.1 (34.6–52.9)	0.753

a mmol/l.

b 10^2^ μmol/mmol of cholesterol. Values are mean ± SD or median (IQR). Groups were compared using the *t-*test and Mann-Whitney *U* test.

c P < 0.05 vs. respective values in the no CAD group. Serum and lipoprotein lipids were analyzed enzymatically, but serum cholesterol, together with noncholesterol sterols, was analyzed by gas-liquid chromatography. Levels of noncholesterol sterols were assessed on a log_10_ scale.

### The impact of cholesterol metabolism on CAD events

By the time of the end of the follow-up period (median, 9.5 [IQR, 8.9–10.0] years), nonfatal or fatal CAD events occurred in 63 patients (17.4%), of which 12 patients (6.2%) were in the no CAD group and 51 patients (30.4%) in the ACS group (*P* < 0.001) (Table 4). Fatal CAD occurred in 32 patients (8.8%) during the follow-up, of which 4 patients (2.1%) were in the no CAD group and 28 patients (16.7%) in the ACS group (*P* < 0.001).

**Table 4:** All-cause mortality and combined nonfatal and fatal CAD events in the study population and separately in the no CAD and ACS groups during the follow-up for a median of 9.5 years

Variables	Study population, n = 363	No CAD, n = 195	ACS, n = 168	*P*
	n (%)			
All-cause mortality				
Alive	291 (80.2)	161 (82.6)	130 (77.4)	0.270
Dead	72 (19.8)	34 (17.4)	38 (22.6)	
CAD, nonfatal and fatal				
No event	300 (82.6)	183 (93.8)	117 (69.6)	<0.001
Event	63 (17.4)	12 (6.2)	51 (30.4)	
CAD, fatal				
Alive	331 (91.2)	191 (97.9)	140 (83.3)	<0.001
Dead	32 (8.8)	4 (2.1)	28 (16.7)	

Groups were compared using chi^2^ test.

The roles of cholesterol absorption efficiency, statin treatment with good adherence (>80%) during the follow-up, gender, and the concentrations of TG, LDL-C, and HDL-C on the CAD outcomes were studied by using the Cox regression model with age at follow-up time separately in the no CAD group and ACS group (Table 5). The adjusted models for CAD events included high cholesterol absorption efficiency, statin treatment with good adherence, HDL-C and gender, LDL-C and gender, and TG and LDL-C. The outcomes were combined nonfatal and fatal events of CAD.

**Table 5:** Results from Cox regression models for CAD outcomes

No CAD		
Variable	Univariate	
	Hazard ratio (95% CI)	*P*
High cholesterol absorption efficiency	1.40 (0.44–4.41)	0.566
Statin treatment after angiography; good adherence for the whole follow-up period	0.61 (0.13–2.77)	0.519
Serum TG, mmol/L	1.87 (0.92–3.84)	0.086
LDL-C, mmol/L	2.00 (0.98–4.04)	0.055
HDL-C, mmol/L	1.15 (0.41–3.28)	0.788
Female	0.47 (0.14–1.57)	0.221

ACS								
Variable	Univariate		Adjusted 1		Adjusted 2		Adjusted 3	
	Hazard ratio (95% CI)	*P*	Hazard ratio (95% CI)	*P*	Hazard ratio (95% CI)	*P*	Hazard ratio (95% CI)	*P*
High cholesterol absorption efficiency	2.36 (1.32–4.20)	0.004	2.70 (1.42–5.13)	0.002	2.42 (1.27–4.61)	0.007	2.41 (1.27–4.58)	0.007
Statin treatment after angiography; good adherence for the whole follow-up period	0.43 (0.25–0.76)	0.003	0.35 (0.19–0.64)	0.001	0.39 (0.21–0.71)	0.002	0.38 (0.21–0.71)	0.002
Serum TG, mmol/L	0.79 (0.46–1.36)	0.397					1.10 (0.64–1.91)	0.726
LDL-C, mmol/L	0.52 (0.35–0.76)	0.001			0.57 (0.39–0.84)	0.004	0.56 (0.37–0.84)	0.005
HDL-C, mmol/L	0.52 (0.19–1.42)	0.200	0.43 (0.14–1.26)	0.124				
Female	1.26 (0.65–2.41)	0.493	1.32 (0.61–2.88)	0.482	1.04 (0.50–2.17)	0.908		

Good statin adherence, adherence rate ≥80%. Age was used as the follow-up time. The adjusted models for the ACS group include both high cholesterol absorption efficiency and statin usage during the follow-up, along with *1*) HDL-C and gender, *2*) LDL-C and gender, and *3*) TG and LDL-C.

In the no CAD group, none of the variables of the univariable model associated with the CAD outcomes (Table 5), possibly due to the low number of events (n = 12) (Table 4). In the ACS group, high cholesterol absorption efficiency increased the CAD outcomes in the univariate and in the adjusted models, whereas good compliance with statin treatment and low LDL-C concentrations were significantly associated with good prognosis. Thus, the triad of cholesterol absorption, compliance of statin treatment, and LDL-C concentration were the essential variables affecting the prognosis in the ACS patients of this study population.

**Fig. 4:**
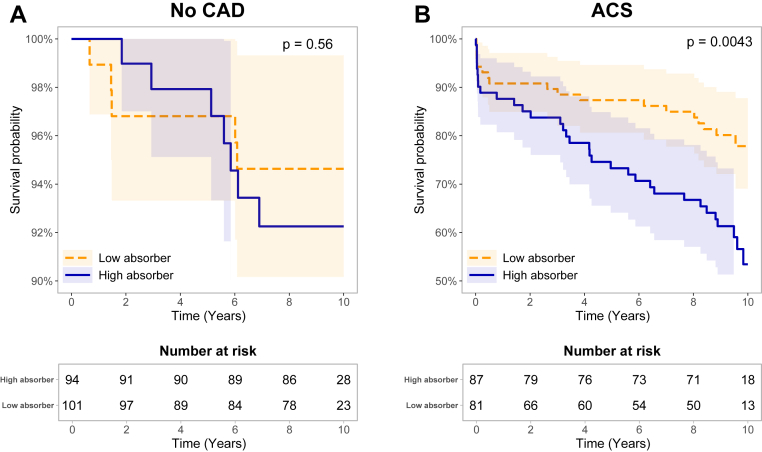
Kaplan-Meier plots for combined nonfatal and fatal CAD events in the no CAD group (A) and ACS group (B) to investigate survival probability in the low versus high cholesterol absorption efficiency subgroups. Differences between the groups were tested by using the log-rank test. The shaded area represents the 95% CI.

We also assessed whether low versus high cholesterol absorption efficiency was related to the rate of the survival probability of CAD in the whole study population divided into the no CAD group and ACS group (Fig. 4). In the no CAD group (panel A), there was no difference in the survival probability between the low and high cholesterol absorbers (*P* = 0.56). In the ACS group (panel B), individuals with high cholesterol absorption efficiency showed the lowest survival rates, whereas those with low cholesterol absorption had the best prognosis (*P* = 0.0043).

## Discussion

The novel findings in this Corogene substudy were, first, that using the Cox weighted regression model, high cholesterol absorption efficiency in the ACS group was associated with the combined nonfatal and fatal CAD outcomes. Statin treatment during the follow-up, with good adherence of >80% as well as low LDL-C concentration, lowered the hazard for CAD outcomes. In the no CAD group, there were no significant associations between these variables and the combined nonfatal and fatal CAD outcomes, possibly due to the low number of events in this subgroup (only 12/192 had a new CAD).

Second, individuals with low cholesterol absorption efficiency had the best probability of survival during the 9.5-year follow-up period. Individuals with high cholesterol absorption efficiency had the worst prognosis. These results confirmed our hypothesis that differences in atherogenicity are also related to whole-body cholesterol metabolism and especially to high cholesterol absorption. These results also confirm earlier suggestions that high cholesterol absorption efficiency may have atherogenic potential (7, 8, 9, 10, 11, 12, 13).

Even though cholesterol absorption efficiency did not differ between the no CAD and the ACS groups, serum biomarkers of cholesterol synthesis were higher in the no CAD versus the ACS group, suggesting more effective cholesterol synthesis and lower cholesterol absorption in the no CAD versus the ACS group, in line with the homeostasis of cholesterol metabolism. Accordingly, high cholesterol absorption is associated with low cholesterol synthesis, low reverse cholesterol transport from the tissues, and low biliary cholesterol elimination from the body to the feces, suggesting that the higher the level of cholesterol absorption and the lower the rates of cholesterol synthesis and biliary cholesterol elimination, the more probable is the risk of atherosclerosis (8, 9, 10, 11).

Fourth, statins decreased serum and LDL-C concentrations in the low and high cholesterol absorbers in the no CAD group and the ACS group. They also decreased cholesterol synthesis in the no CAD low and high absorbers as well as in the ACS low absorbers but had no effect on cholesterol synthesis in the ACS high absorbers. It has been demonstrated earlier that statins also reduce biliary cholesterol elimination from the body (28, 29). For example, atorvastatin (80 mg/day) reduced mean biliary cholesterol elimination by 46% (SEM 3.0%, *P* < 0.05) and increased cholesterol absorption efficiency by 103% (SEM 1.0%, *P* < 0.05) (29).

Because elevated LDL-C concentration causes ASCVDs, and lowering it is the cornerstone in prevention of the development and event risks of ASCVDs (5, 30, 31), the question arises of whether high cholesterol absorption efficiency should be acknowledged in prevention of the development of ASCVDs. Cholesterol absorption efficiency and cholesterol synthesis can be screened by assays of serum cholesterol absorption and synthesis biomarkers. High cholesterol absorption can also be screened with the risk alleles of the intestinal ABCG5 and ABCG8 transporters. Thus, it is possible to diagnose the profiles of cholesterol absorption efficiency and cholesterol synthesis from a blood sample and take advantage of this information together with LDL-C concentrations to prevent the development and event risks of ASCVDs.

Ezetimibe decreases cholesterol absorption efficiency. Therefore, the treatment schedule for high cholesterol absorbers may have similarities to the recent lipid-lowering recommendations to patients at very high ASCVD risk (30, 31). These recommendations include a proposal to start with a combination therapy of statin + ezetimibe, or alternatively, in statin intolerance, proprotein convertase subtilisin kexin type 9 inhibitors, proprotein convertase subtilisin kexin type 9 siRNA (inclisiran), or bempedoic acid, which can replace statin and be combined with ezetimibe. All these drugs upregulate LDL receptor expression in the liver and lower circulating LDL-C concentrations.

Ezetimibe inhibits the absorption of cholesterol by 30% (SEM, 4.3%; *P* < 0.0001), increases biliary cholesterol elimination by 67% (SEM, 12%; *P* < 0.0001), and decreases LDL-C concentrations by 20% (SEM, 2%; *P* < 0.0001) (32). If ezetimibe is combined with dietary phytosterols, which also decrease cholesterol absorption, their combination intensifies the positive effects; the combination of ezetimibe and phytosterol consumption relative to the consumption of ezetimibe alone significantly and additionally reduced cholesterol absorption efficiency by 27%, increased biliary elimination of cholesterol by 24%, and reduced LDL-C concentrations by 7% (33). Thus, high cholesterol absorbers would need ezetimibe alone or in combination with phytosterols as a basis to reduce cholesterol absorption efficiency and also ezetimibe combined with other LDL-C-lowering drugs, as needed.

The positive correlation between cholesterol absorption efficiency and the risk of CAD outcomes suggests that the higher the level of cholesterol absorption and the lower the rates of cholesterol synthesis and biliary cholesterol elimination, the more probable the risk of atherosclerosis (10, 32, 34). This can be mitigated by diagnosing the profile of high cholesterol absorption efficiency as early as possible and providing cholesterol absorption inhibitors combined with statins or other LDL-C-lowering drugs as needed.

### Limitations of the study

Because of the size of the study population (363 individuals), and the laborious methodology involved in the investigation of the overall cholesterol metabolism at both population and laboratory levels, cholesterol metabolism could be evaluated only by means of the relative methods, using validated serum biomarkers of cholesterol synthesis and cholesterol absorption efficiency. For this reason, it was not possible to obtain information on biliary cholesterol elimination from the body, which, together with cholesterol synthesis, inversely correlates with the risk of atherosclerosis. However, the relative levels of serum biomarkers were useful and appropriate, and the study population was in a steady state, which is essential in metabolic studies. Since serum biomarkers of cholesterol synthesis and absorption efficiency have their individual metabolic pathways, it seems important to use more than one biomarker for both synthesis and absorption to verify the results. The second limitation related to the size of the study population probably reflects the finding that in the Cox regression model in the no CAD group, none of the variables of the univariate model associated with the CAD outcomes, possibly due to the low number of events.

## Conclusions

In this Corogene substudy with the ∼10-year follow-up period, high cholesterol absorption efficiency was associated with combined nonfatal and fatal CAD events in the ACS group. Individuals with high cholesterol absorption efficiency also showed the worst probability of survival, whereas those with low cholesterol absorption efficiency showed the best prognosis. These results confirmed the hypothesis that high cholesterol absorption efficiency has atherogenic potential. Individuals with high cholesterol absorption need a combination of lipid-lowering therapy with cholesterol absorption inhibition combined with LDL-C-lowering drugs when needed to modify cholesterol metabolism to a less atherogenic state.

## Data Availability

The data are available by request from J.S.

## Supplemental Data

This article contains supplemental data.

## Conflict of Interest

J. S. has received grants from Lecture honoraria: Abbott, Amgen, Amarin, AstraZeneca, Bristol Myers Squibb, and Novo-Nordisk. All other authors declare that they have no conflicts of interest with the contents of this article.
