# Supplementary material for: High cholesterol absorption efficiency increases the risk of the nonfatal and fatal atherosclerotic events
Source: J Lipid Res. 2025 Dec 30;67(2):100974. doi: 10.1016/j.jlr.2025.100974 (PMC12873726; doi:10.1016/j.jlr.2025.100974)
Supplement: Supplemental Table S1 [file mmc1.docx]

SUPPLEMENTAL TABLE S1. Baseline clinical characteristics in the study population without CAD and with ACS and further divided into subgroups of low and high cholesterol absorbers.

| **Variables** | **Study population, n = 363** | **No CAD,  Low absorbers n = 94** | **ACS,  Low absorbers n = 87** | ***P-*value No CAD vs ACS Low absorbers** | **No CAD,  High absorbers n = 101** | **ACS,  High absorbers n = 81** | ***P*-value  No CAD vs ACS High absorbers** | **Missing, n (%)** |
| --- | --- | --- | --- | --- | --- | --- | --- | --- |
| Men/women, n (%) | 187/176 (52/48) | 40/54 (43/57) | 54/33 (62/38) | **0.013** | 57/44 (56/44) | 36/45 (44/56) | 0.145 |  |
| Age, years | 62.1 ± 3.0 | 61.7 ± 1.3 | 61.8 ± 4.2 | 0.920 | 61.9 ± 1.2 | 63.1 ± 4.1 | **0.014** |  |
| Weight, kg | 78.1 ± 17.5 | 80.4 ± 18.4 | 80.5 ± 17.4 | 0.953 | 78.1 ± 18.2 | 72.7 ± 14.6 | **0.026** | 1 (0) |
| Body mass index, kg/m2 | 26.9 ± 5.1 | 27.8 ± 4.9 | 27.9 ± 5.1 | 0.935 | 26.3 ± 5.5 | 25.5 ± 4.2 | 0.285 | 2 (1) |
| *Atherosclerotic risk factors* | |  |  |  |  |  |  |  |
| Dyslipidaemia, n (%) | 213 (59) | 53 (56) | 56 (64) | 0.345 | 53 (52) | 51 (63) | 0.204 |  |
| Hypertension, n (%) | 214 (59) | 57 (61) | 49 (56) | 0.661 | 57 (56) | 51 (63) | 0.460 |  |
| T1D or T2D | 52 (14) | 11 (12) | 11 (13) | 1.000 | 13 (13) | 17 (21) | 0.206 |  |
| Current smoker, n (%) | 117 (32) | 36 (38) | 19 (22) | **0.025** | 42 (42) | 20 (25) | **0.034** |  |
| Ex- smoker, n (%) | 103 (29) | 13 (14) | 37 (43) | **<0.001** | 20 (20) | 33 (42) | **0.002** |  |
| *Essential standard laboratory measurements* | | |  |  |  |  |  |  |
| Blood haemoglobin, g/L | 138 ± 16 | 141 ± 11 | 139 ± 12 | 0.211 | 138 ± 17 | 132 ± 18 | 0.057 | 37 (10) |
| Blood HbA1c, mmol/mol | 6.3 ± 1.2 | 7.1 ± 1.7 | 6.1 ± 0.9 | 0.314 | 6.9 ± 1.7 | 6.4 ± 1.2 | 0.469 | 292 (80) |
| Plasma creatinine, µmol/L* | 74 (64 - 87) | 77 (65 - 91) | 71 (63 - 81) | **0.015** | 76 (67 - 93) | 70 (61 - 84) | 0.053 | 68 (19) |
| Serum TSH, mU/L* | 1.93 (1.33 - 3.04) | 1.92 (1.33 - 3.15) | 1.80 (1.10 - 2.94) | 0.664 | 1.94 (1.18 - 3.48) | 1.94 (1.48 - 2.57) | 0.713 | 165 (45) |
| Plasma ASAT, U/L* | 38 (28 - 52) | 33 (32 - 34) | 42 (27 - 64) | 0.175 | 32 (24 - 38) | 44 (30 - 74) | **0.034** | 276 (76) |
| Plasma NT-proBNP, ng/L* | 366 (155 - 1377) | 208 (110 - 460) | 405 (201 - 1554) | **<0.001** | 380 (159 - 1450) | 706 (265 - 2982) | **0.020** | 24 (7) |
| Plasma NT-proBNP >= 600 ng/L | 133 (39) | 20 (22) | 35 (45) | **0.002** | 38 (38) | 40 (57) | **0.021** |  |
| Plasma NT-proBNP >= 900 ng/L | 110 (32) | 13 (14) | 31 (40) | **<0.001** | 35 (35) | 31 (44) | 0.288 |  |
| Serum hs-CRP, mg/L* | 2.3 (1.0 - 8.6) | 1.6 (0.9 - 3.9) | 4.0 (1.3 - 20.6) | **<0.001** | 1.6 (0.7 - 4.8) | 6.2 (1.4 - 31.7) | **<0.001** |  |
| *S Serum and lipoprotein lipids, mmol/L* | |  |  |  |  |  |  | 19 (5) |
| Serum cholesterol | 4.84 ± 1.02 | 4.89 ± 0.95 | 4.95 ± 0.89 | 0.700 | 4.71 ± 1.11 | 4.83 ± 1.08 | 0.469 |  |
| LDL-C | 2.92 ± 0.85 | 2.79 ± 0.71 | 3.21 ± 0.80 | **<0.001** | 2.74 ± 0.90 | 3.03 ± 0.90 | **0.039** |  |
| HDL-C | 1.38 ± 0.45 | 1.52 ± 0.54 | 1.25 ± 0.29 | **<0.001** | 1.43 ± 0.48 | 1.27 ± 0.38 | **0.018** |  |
| Serum triglycerides | 1.31 ± 0.62 | 1.36 ± 0.71 | 1.37 ± 0.49 | 0.954 | 1.18 ± 0.60 | 1.37 ± 0.65 | 0.061 |  |
| *St Statin treatment before angiography with good adherence* | | |  |  |  |  |  |  |
| Statins, no, n (%) | 261 (72) | 59 (63) | 74 (85) | **0.001** | 65 (64) | 63 (78) | 0.071 |  |
| Statins, yes, n (%) | 102 (28) | 35 (37) | 13 (15) |  | 36 (36) | 18 (22) |  |  |
| *Statin treatment after angiography; good adherence for the whole follow-up period* | | | |  |  |  |  |  |
| Statins, no, n (%) | 221 (61) | 74 (79) | 39 (45) | **<0.001** | 75 (74) | 33 (41) | **<0.001** |  |
| Statins, yes, n (%) | 142 (39) | 20 (21) | 48 (55) |  | 26 (26) | 48 (59) |  |  |

Mean ± SD, n (%). Groups were compared using t-test, Mann-Whitney *U* test, and chi^2^ test. *Values shown are median (IQR) and tested using Mann-Whitney *U* test. Abbreviations: ACS = acute coronary syndrome, ASAT = aspartate aminotransferase, CAD = coronary artery disease, HbA1c = haemoglobin A1c, hs-CRP = high sensitive C-reactive protein, IQR = interquartile range, NT- proBNP = N-terminal natriuretic peptide, TSH = thyroid-stimulating hormone. Serum and lipoprotein lipids were analysed enzymatically. Statin treatment: simvastatin, atorvastatin, or rosuvastatin. Regarding the age of this study population, the optimal plasma cutoff of NT-proBNP diagnosing heart failure is > 900 ng/L.
